# Unraveling the Impact of Blended Learning vs. Online Learning on Learners’ Performance: Perspective of Self-Determination Theory

**DOI:** 10.3390/bs15091263

**Published:** 2025-09-16

**Authors:** Qing Yu, Kun Yu, Jiyao Wang

**Affiliations:** 1 Institute of Higher Education, Fudan University, Shanghai 200433, China; 2429560977@qq.com; 2 School of Social Development and Public Policy, Fudan University, Shanghai 200433, China

**Keywords:** blended learning, online learning, learning performance, meta-analysis

## Abstract

After the COVID-19 pandemic, online and blended learning (BL) have been very popular worldwide. They have become as important as face-to-face (F2F) learning. Previous meta-analyses examined the effects of BL and online learning (OL) compared to F2F learning. However, there is no meta-analytic evidence on the effects of BL vs. OL. Which is more effective: BL or OL? So, this study compares the impact of BL and OL based on 37 empirical articles (2000–2024) via meta-analysis. The results suggest that BL has a positive upper-medium effect on student learning outcomes (*SMD* = 0.611, *p* < 0.001), especially on cognitive outcomes (*SMD* = 0.698, *p* < 0.001) and affective outcomes (*SMD* = 0.533, *p* < 0.001). Moreover, moderator analysis finds that BL’s effects are better than OL (1) for a class size of 0~50 students (2) for K-12 and university students (3) within 3 months of intervention (4) on non-STEM subjects (5) with different teachers (6) with 30%~69% proportion of OL (7) using mixed interaction (8) with mixed and group learning (9) on Asian students. Moreover, the results provide valuable suggestions for educators and researchers to improve BL’s practices.

## 1. Introduction

Traditional face-to-face (F2F) learning is inflexible, with time constraints, high travel costs, and limited learning opportunities (Khatony et al., 2009; Zhang et al., 2015). Online and BL emerged to overcome these drawbacks of F2F learning, and they have become a worldwide trend (Werth et al., 2013). OL refers to learners accessing the internet via different devices (such as smartphones, laptops, etc.) for synchronous or asynchronous learning (Dhawan, 2020). Unlike F2F and OL, BL is the integration of both (Graham et al., 2013). Recently, the global COVID-19 pandemic and increasing displacement have forced many countries to rapidly accelerate and transition to online and blended forms of learning (Greenhow et al., 2022). Subsequent digital transformation has also driven this significant shift (T. T. Wu et al., 2024). A recent report from EDUCAUSE emphasizes embracing and improving blended and online learning (Muscanell, 2024). Previous studies reveal that OL and BL are generally more powerful than F2F learning (Schmid et al., 2023; Topping et al., 2022). Previous meta-analyses mainly analyze the effects of BL and F2F, with less attention given to the effects of BL and OL. Thus, which is more effective, BL or OL? Currently, there is no consensus on this in the existing experimental or quasi-experimental research. In addition, there is no meta-analytic evidence to clarify it. Therefore, this study aims to compare the effects of BL vs. OL on student learning performance through meta-analysis and to examine the influence of some moderator variables.

## 2. Literature Review

### 2.1. Self-Determination Theory

Self-determination theory (SDT) was proposed by Deci and Ryan (1985). This theory argues that people have three basic psychological needs: autonomy, competence, and relatedness. Autonomy means that we need to feel in control of our behaviors and goals; competence signifies the need to feel effective, capable, and challenged; and relatedness embodies the need to engage in interactions, feel connected, and care for others (Ryan & Deci, 2017, 2020). When instruction adequately fulfills those psychological needs, students will be motivated to engage in learning activities (Hsu et al., 2019). Learners have basic psychological needs in online and blended learning environments. SDT argues that optimal growth occurs when three basic psychological needs are satisfied (Ryan & Deci, 2017). Students value the role of face-to-face activities (Vanslambrouck et al., 2018), but pure OL lacks this component while BL does. Relatedness is critical in OL (Chiu, 2022), but BL can better satisfy students’ relatedness than OL due to the face-to-face interactions between peers and teachers.

### 2.2. OL and BL

OL was first used in 1995 (Singh & Thurman, 2019). Simply, it refers to the use of the Web to obtain relevant learning materials during learning (Ally, 2004). OL is also named distance learning, e-learning, or Web-based learning (Ally, 2004; Singh & Thurman, 2019). OL has many advantages, which may help to bridge the digital divide (L. Ma & Lee, 2021) and provide various education opportunities. Nevertheless, OL also has some shortcomings, such as insufficient peer contact and social interaction (J. H. Wu et al., 2010; Peng & Fu, 2021), isolation and disconnectivity (L. Ma & Lee, 2021), difficulties for students to manage time and keep self-motivation (Chou & Chou, 2011), and difficulty learning in depth (Holzweiss et al., 2014). So, OL may not always be effective for promoting student learning performance. For instance, Kim and Kim’s (2023) meta-analysis suggested that OL did not significantly improve students’ knowledge achievement and learning attitude during the COVID-19 pandemic.

To address the drawbacks of OL, BL emerged (Bonk & Graham, 2012; Bozkurt, 2022; Ustun & Tracey, 2020) in the early 2000s and has been very popular in education (Bozkurt, 2022; Ghimire, 2022; Rasheed et al., 2020). BL is the integration of online and F2F learning (Graham et al., 2013; Castro, 2019). However, BL does more than just add F2F learning to OL (Keengwe & Kang, 2013). BL is a branch of OL (Asarta & Schmidt, 2020), also called flexible, mixed, or hybrid learning (Garrison & Kanuka, 2004; Tayebinik & Puteh, 2013; Smith & Hill, 2019). Facilitating flexibility is one of the benefits of BL (Müller et al., 2023). BL’s flexibility maximizes many positive education functions (Dziuban et al., 2018). Wang and Raman’s (2025) review revealed that BL was more effective in promoting student academic performance, learning motivation, and satisfaction compared to F2F learning. Compared to OL, BL can foster students’ self-regulation ability (Alharthi & Elsigini, 2022), satisfaction, interaction, engagement, perceived flexibility, intrinsic motivation, self-efficacy, and learning achievement (Finlay et al., 2022; L. Ma & Lee, 2021; Su et al., 2021; N. T. T. Thai et al., 2017; Zhan et al., 2017). Recent studies also suggested that students prefer BL to OL (Arain et al., 2022; Nasution et al., 2021). In the post-COVID-19 pandemic era, educators can use BL to reach optimal learning engagement and student satisfaction (Suriagiri et al., 2022).

### 2.3. Effectiveness of BL vs. OL

So, is BL more effective than OL? Many studies compare the effects of BL and F2F learning, but few compare BL and OL. Meanwhile, for the latter, they have yet to reach an agreement; the results can be divided into three types. The details are as follows.

(1) BL is better than OL (e.g., Alqahtani, 2010; Al-Qahtani & Higgins, 2013; Alipour, 2020; Ali et al., 2023; Alzahrani, 2022; Bicen et al., 2014; Bock et al., 2021; Caldwell, 2006; Chao et al., 2021; Dousti & Amirian, 2023; Grant, 2016; Haftador et al., 2021; Khotimah et al., 2022; McCutcheon et al., 2018; L. Ma & Lee, 2021; Ranjan, 2018; Sidorova et al., 2022; Terry et al., 2016; N. T. T. Thai et al., 2020; Zhan et al., 2017), e.g., Dousti and Amirian (2023) conducted a true experiment where they found that BL can significantly improve students’ English writing achievement compared to OL. L. Ma and Lee (2021) used a randomized controlled experiment and found that BL can significantly improve students’ satisfaction compared to OL. Ali et al. (2023) also revealed that BL and OL can significantly increase students’ grammar performance, but BL’s effect is significantly higher than OL.
(2) There is no significant difference between BL and OL (e.g., Alonso et al., 2010; Caldwell, 2006; Fife, 2020; J. Lim et al., 2008; Moradimokhles & Hwang, 2022; Paul et al., 2023; Sezer & Esenay, 2022; T. Thai et al., 2015; Xin et al., 2015; Yen et al., 2018), e.g., Sezer and Esenay (2022) conducted a quasi-experiment to reveal that flipped classrooms had no significant impact on nurse students’ academic performance and critical thinking compared to OL; Moradimokhles and Hwang (2022) used an experiment to determine if BL can develop nursing students’ English language skills compared to OL, but the effect is insignificant.
(3) OL is better than BL (e.g., Bock et al., 2021; Charytanowicz et al., 2024; Gundlach et al., 2015; Larson & Sung, 2009; Paul et al., 2023; Sizemore et al., 2024; Taylor et al., 2023), e.g., Bock et al. (2021) used a randomized study to find that BL cannot promote students’ clinical skills and is even inferior to OL; Paul et al. (2023) found that BL did not improve medical students’ academic performance, and its impact was worse than OL.

Overall, though BL has become popular worldwide, our knowledge of effective BL has lagged behind practice (Pulham & Graham, 2018). The ambiguous effect of BL compared to OL creates confusion for educational practitioners and teachers.

### 2.4. Past Meta-Analysis and Research Gaps

Various meta-analyses examine the effects of BL. Some studies focus on different educational levels, e.g., K-12 (Li & Wang, 2022), higher education (Bernard et al., 2014; Müller & Mildenberger, 2021; Vo et al., 2017), and K-12 to higher education (Means et al., 2013; Yu et al., 2025). Some focus on varying disciplines, e.g., teacher education (K. Ma et al., 2023; Schmid et al., 2023), medical education (Vallée et al., 2020), and nurse education (Du et al., 2022). Moreover, a meta-analysis also compares the effectiveness of BL across different countries (Cao, 2023). These studies find that BL is more effective or has the same effect as F2F learning. However, all of them only compared the effectiveness of BL and F2F learning, ignoring the comparison of BL and OL.

This meta-analysis aims to compare the effectiveness of BL vs. OL. We try to solve the research gaps as follows. First, the meta-analytic evidence on the effects of BL compared to pure OL is limited (Ranjan, 2018; Zhan et al., 2017). Prior research neglected to compare BL and OL, lacking systematic quantitative synthesis (i.e., meta-analysis) to provide robust evidence about the comparative effect of BL and OL. Second, after the COVID-19 pandemic, the importance of BL has further increased (Wang & Raman, 2025), and the challenge is to ensure an effective blending of F2F and OL (Hill & Smith, 2023). BL will be an effective method if a balance is formed between online and F2F learning (Ustun & Tracey, 2021). So how do we find this balance? In other words, what variables influence the effects of BL?

The success of technologies depends on educators’ abilities to analyze their educational merit, affordances, and constraints to strategically repurpose them for educational contexts (Ali et al., 2023; Bower & Sturman, 2015). Conducting a meta-analysis study to reveal the actual effects of BL and moderators is necessary and valuable for educators and researchers.

### 2.5. Possible Moderators Influencing Effectiveness of BL vs. OL

BL’s effectiveness depends on the study design and BL’s designs. We selected 12 moderators based on BL characteristics and prior meta-analyses (Yu et al., 2025). These moderators may affect the effectiveness of BL vs. OL on students’ learning outcomes.

**Class Size:** Prior studies argue that smaller class sizes (e.g., 50 or below) are more beneficial for student learning (Glass & Smith, 1979; Li & Wang, 2022; Shin & Chung, 2009). Different class sizes can affect interactions and student learning, so, it may moderate BL’s effects (Du et al., 2022; Li & Wang, 2022; Yu et al., 2025).

**Grade Level:** It refers to the students’ grade level. There are differences between university and K-12 students in cognitive abilities, and students’ acceptance and attitude toward BL may vary by grade levels (Yu et al., 2025). So, it is a potential moderator (Du et al., 2022; Li & Wang, 2022; Schmid et al., 2023).

**Learning Duration:** Prior meta-analyses found that the influence of learning durations on BL’s effects is mixed, e.g., longer is better (Bernard et al., 2014), or shorter is better (Du et al., 2022; Vo et al., 2017). Hence, it may be a moderator (Li & Wang, 2022; Vo et al., 2017; Yu et al., 2025).

**Subject:** BL’s effects may vary by subject. Owston et al. (2020) found that students in STEM subjects achieved significantly better performance than students in non-STEM subjects. Differences between subjects should be considered (Becher, 1994). So, the subject is a potential moderator (Vo et al., 2017; Schmid et al., 2023; Yu et al., 2025).

**Teacher:** Teacher quality matters most in influencing student achievement (Goldhaber, 2016). Their expertise and technological literacy can affect student learning (Darling-Hammond, 2000). So, it is a potential moderator (Li & Wang, 2022; Means et al., 2013; Vo et al., 2017).

**Region:** It refers to the place where the study was conducted. There are differences in culture, social economics, and education systems between different regions (Yu et al., 2025). So, the region is a potential moderator (Li & Wang, 2022; Schmid et al., 2023).

**Proportion of Online Learning (POL):** It is the percentage of overall learning time spent online. How many learning activities will be online is critical. POL affects BL’s effects on student learning (Means et al., 2013; Yu et al., 2025). So, POL is a potential moderator (Owston & York, 2018; Yu et al., 2025).

**Type of Online Interaction (TOI):** It is the type of online communication (Yu et al., 2025), e.g., synchronous and asynchronous. Different TOIs have both advantages and disadvantages and may result in different learning effects. So, TOI may moderate BL’s effects on student learning (Li & Wang, 2022; Means et al., 2013; Yu et al., 2025).

**Online Learning Activity (OGA):** It is the type of OL activity, i.e., group or individual online activities (Yu et al., 2025). OGA could affect student learning (Chen et al., 2018; Li & Wang, 2022). Namely, it is a potential moderator influencing BL’s effects (Li & Wang, 2022; Yu et al., 2025).

**Publication Year:** It is the published year of the literature. The publication year reflects the technological advancements and developments behind BL. In other words, publication year is a possible moderator that moderates BL’s effects (Du et al., 2022; Vo et al., 2017; Yu et al., 2025).

**Publication Type:** It is the published type of the literature, e.g., journals, dissertations, and conferences. Different publication types may have different preferences, e.g., journal articles tend to report larger effects (Cheng et al., 2019; Vo et al., 2017). So, publication type may moderate BL’s effects (Vo et al., 2017).

### 2.6. Purpose

**Figure 1:**
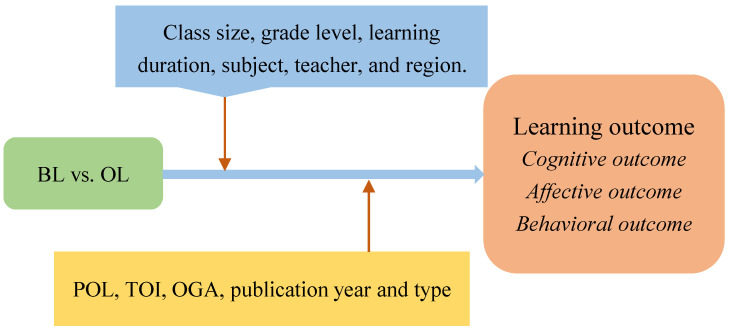
Research framework.

BL combines the merits of F2F and online activities and it may outperform OL (Keengwe & Kang, 2013). So, is this true? Figure 1 displays the research framework. This study aims to offer evidence-based answers to the following questions.

RQ1. What is the overall effect size (ES) of BL vs. OL?

RQ2. How do potential moderators impact BL’s effects, e.g., class size, grade level, learning duration, subject, teacher, region, POL, TOI, TOGA, and publication type and year?

## 3. Methods

Meta-analysis is a mathematical procedure that averages results across several similar studies (Andrade, 2020). This meta-analysis is conducted by the following stages: (1) literature search, (2) literature selection, (3) variable coding, (4) calculating the ES, and (5) moderator analysis (Field & Gillett, 2010).

### 3.1. Literature Search

Several main literature sources (i.e., Web of Science Core Collection and Scopus) are used to search target papers. Those databases are high quality. The search terms are connected based on Boolean operators to retrieve the relevant literature. Specifically, terms as follows are connected (“blended learning” or “blended instruction” or “blended teaching” or “hybrid learning” or “hybrid instruction” or “hybrid teaching” or “mixed learning” or flip*) AND (“online learning” or “online education” or “online teaching” or “e-learning” or “distance learning”) AND (“learning performance” or “learning outcome*” or “academic achievement*” or “learning achievement*” or “academic outcome*” or “academic performance”) AND (learner* or student*) AND (treatment* or intervention* or experiment*). The search time interval is limited to Jan 2000 to Nov 2024. After excluding the irrelevant literature (e.g., review, correction, editorial, etc.), we retrieve 538 articles (WOS = 191, Scopus = 347). Later, after removing the duplicates, 376 articles remained.

### 3.2. Literature Selection and Quality

We selected a study based on the following standards (Table 1).

**Figure 2:**
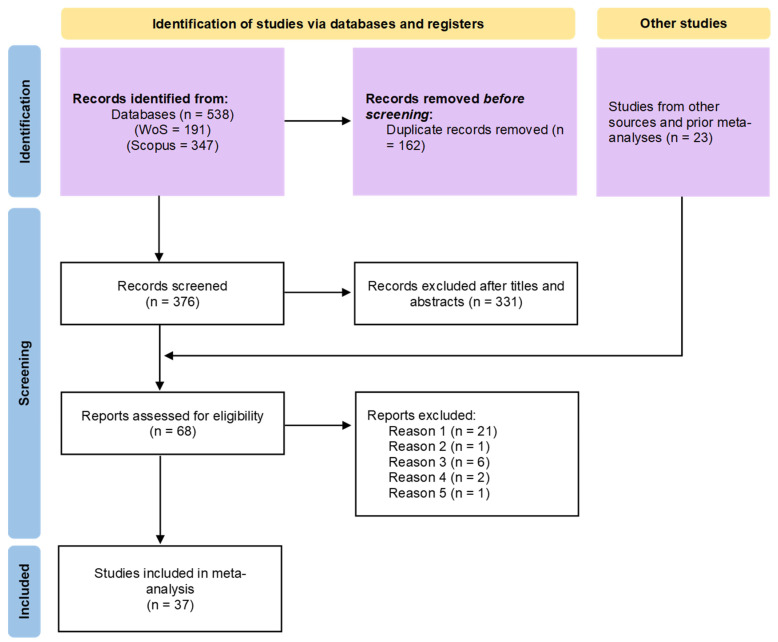
Literature selection.

According to the inclusion and exclusion criteria, 68 papers remained after filtering the titles and abstracts. Next, we read the full text and eliminate 31 papers. Last, we obtain 37 papers. These steps strictly follow the guidelines given by Prisma (Page et al., 2021) (Figure 2).

We assess the study quality independently based on the tool of Kmet et al. (2011). These criteria include research question, sampling, research design, method, result, etc. Each criterion was pointed (“yes” = 2, “partial” = 1, “no” = 0). Most articles exceed 50% of the summary point, indicating acceptable quality.

### 3.3. Variable Coding

Following the methods of Yu et al. (2025) and Li and Wang (2022), we divide the variables into four categories, e.g., dependent variable, background feature, BL design feature, and literature feature. According to the characteristics of BL and prior meta-analyses (Bernard et al., 2014; Li & Wang, 2022; Means et al., 2013; Vo et al., 2017; Yu et al., 2025), we code these variables into different subtypes. The Kappa exceeds 0.85, and the ICC = 1. The details are described in Table 2.

### 3.4. Data Analysis

We use the Comprehensive Meta-Analysis (CMA) 3.0 to calculate the ES and perform moderator analysis (Borenstein et al., 2009). Considering that the literature included in this study is not large, we choose the Standardized Mean Difference (*SMD*) as the ES to quantify the effect of BL versus OL. In addition, we conduct some tests: (1) publication bias: judging whether any literature has been omitted; (2) heterogeneity analysis: determining differences in included literature; (3) sensitivity analysis: ensuring the reliability.

## 4. Results

### 4.1. Publication Bias, Heterogeneity Analysis, and Sensitivity Analysis

**Figure 3:**
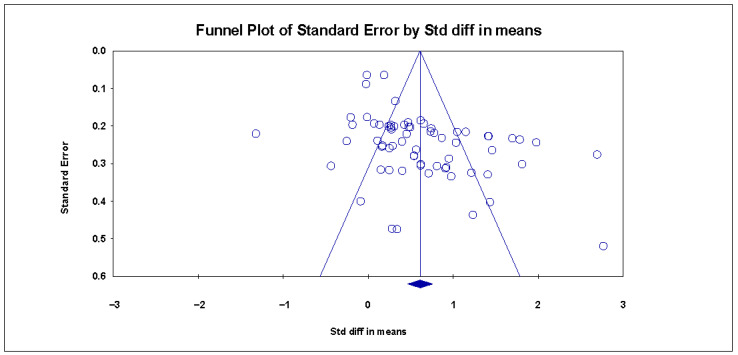
Funnel plot.

**Figure 4:**
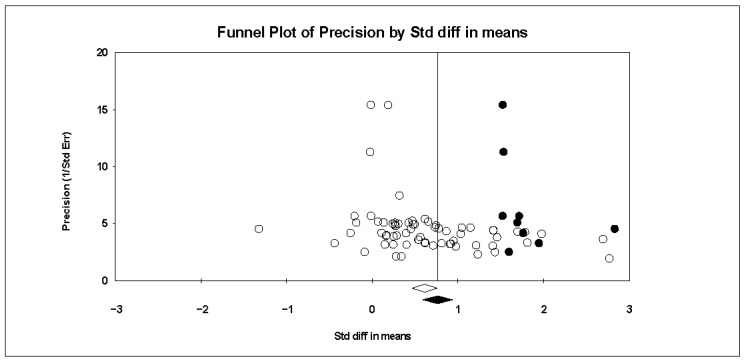
Funnel plot after trim-and-fill.

We selected three methods to test the publication bias. First, the funnel plot is examined. The asymmetrical distribution of ES in the funnel plot indicates the presence of publication bias (Duval & Tweedie, 2000). However, Figure 3 shows that the scatter distribution in the funnel plot is not very even, indicating a potential risk of publication bias. Next, the trim-and-fill method is explored (Greenhouse & Iyengar, 1994). The result shows nine missing studies under the random-effects model (Figure 4). Last, classic fail-safe *Nfs* is examined, and the result shows *Nfs* = 6679, 5*K + 10 = 345 (K = 67); the result is much larger than the comparison standard (*Nfs* > 345) (Rosenthal, 1991). To summarize, the article pool is considered reliable regarding publication bias.

The result shows that the *Q* test (*p* < 0.001) is significant, and *I*^2^ is larger than 75% (Table 3). So, the heterogeneity is considerable. In light of this, we choose the random-effects model to compute the overall ES of BL (Wilson et al., 2020). Moreover, moderator analysis is necessary.

To ensure the reliability, we conducted sensitivity analysis with the one-study-removed method. The result suggests that overall ES, excluding one study, all fall within a reasonable scale [0.466, 0.755], so this meta-analysis is robust.

### 4.2. Characteristics of Studies

This meta-analysis includes 37 true or quasi-experimental studies (with 67 independent ES). The publication year ranges from 2006 to 2024 and comprises 30 journal articles, 5 doctoral dissertations, and 2 conferences. The education level covers K-12 (k = 3), adult (k = 1), and university (k = 32). The included studies were conducted in Asia (k = 21), North America (k = 11), Europe (k = 4), and Australia (k = 1). The subject covers STEM (k = 23) and non-STEM (k = 14). The learning duration includes <1 month (k = 4), 1–3 months (k = 15), and ≥3 months (k = 16). The class size covers ≤30 (k = 15), 30–50 (k = 12), 51–100 (k = 7), and >100 (k = 3). The study design includes the quasi-experiment (k = 21) and true experiment (k = 15).

### 4.3. Overall ES of BL vs. OL

**Figure 5:**
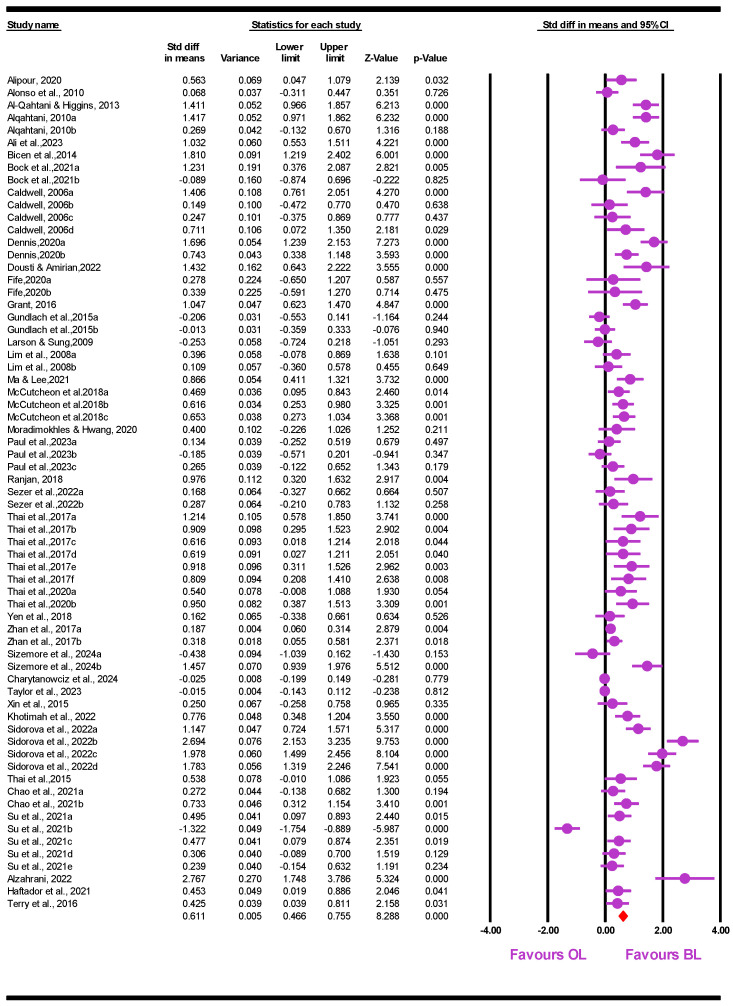
Forest plot.

The result shows that BL has an upper-medium effect on student performance (*SMD* = 0.611, 95% *CI* = [0.466, 0.755], *p* < 0.001). Figure 5 shows the details of each ES.

The result shows that BL has upper-medium effects on affective outcomes and cognitive outcomes, and an insignificant effect on behavioral outcomes (Table 4). The *Q*-between suggests that the learning outcome (*p* < 0.01) has a moderating effect.

### 4.4. Moderator Analysis

**Class size.** The result suggests that BL has upper-medium effects when class size is 31–50 and ≤30 and has a lower-medium effect when class size is 51–100, while BL’s effect is insignificant when class size is >100 (Table 5). The *Q*-between suggests that the class size (*p* < 0.001) significantly moderates the effects of BL.

**Grade level.** The result shows that BL’s effect on K-12 students is large, is upper-medium on university students, and is small on adult learners (Table 5). The *Q*-between suggests that the grade level (*p* < 0.001) moderate BL’s effects.

**Learning duration.** The result shows that <1 month has a large effect and 1–3 months and ≥3 months have upper-medium effects (Table 5). The *Q*-between suggests that the learning duration (*p* > 0.05) is an insignificant moderator.

**Subject.** The result suggests that BL’s effect on non-STEM is large and is lower-medium on STEM (Table 5). The *Q*-between shows that the subject (*p* < 0.001) is a significant moderator.

**Teacher.** The ES is larger when BL and OL use different teachers than the same teachers (Table 5). Therefore, the teacher (*p* < 0.01) is a significant moderator.

**POL.** The result indicates that BL has upper-medium effects when POL is 30–49%, 50%, and 51–69%, has small effect when POL is 70–79%, and has insignificant effects when POL is 80% (Table 6). The *Q*-between suggests that POL (*p* < 0.001) significantly moderates the effects of BL.

**TOI.** The result shows that mixed interaction effect is large, synchronous interaction effect is upper-medium, and asynchronous interaction effect is lower-medium (Table 6). The *Q*-between reveals that TOI (*p* < 0.01) is a significant moderator.

**OGA.** The result reveals that mixed learning has a large effect, group learning has an upper-medium effect, and independent learning has a lower-medium effect (Table 6). The *Q*-between suggests that OGA (*p* < 0.05) is an insignificant moderator.

**Region.** The result shows that BL’s effect on Asian students is large and is upper-small on North American, European, and Australian students (Table 6). The *Q*-between indicates that the region (*p* < 0.05) is a significant moderator.

**Publication type.** The result shows that dissertations’ ES is large, journals’ ES is upper-medium, and conferences’ ES is insignificant (*p* > 0.05) (Table 6). The *Q*-between suggests that the publication type (*p* > 0.05) is an insignificant moderator.

**Figure 6:**
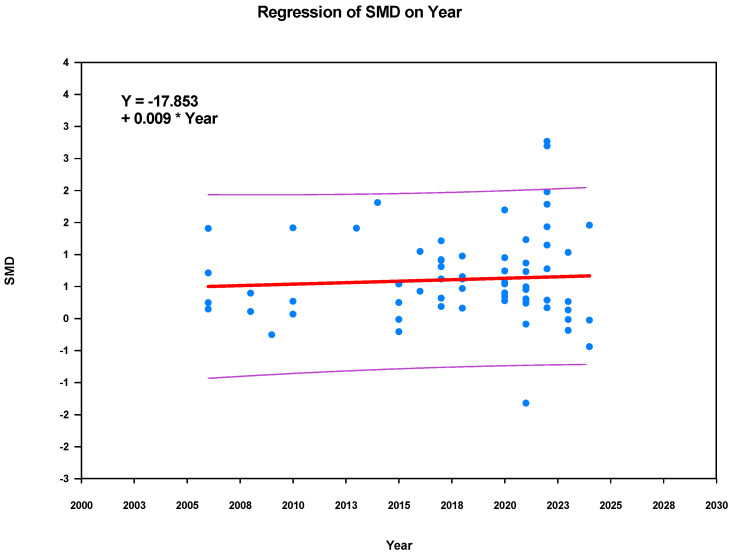
Meta-regression plot.

**Publication year.** The meta-regression indicates that BL’s ES is positive with publication year (*β* = 0.009, *p* = 0.545) (Figure 6). Therefore, it has no moderating effect.

## 5. Discussion

### 5.1. Overall ES of BL vs. OL (RQ1)

These included studies present varying effects of BL vs. OL, e.g., BL is better than OL; there is no significant difference between BL and OL; and OL is better than BL. This meta-analysis tries to compare the effectiveness of BL with OL on learners’ learning based on 67 ES from 37 independent studies. The result suggests that BL has an upper-medium effect on student learning compared to OL. It could be interpreted for several reasons. *First*, BL incorporates the advantages of F2F and OL, and it could alleviate the shortcomings of OL (Bonk & Graham, 2012; Ustun & Tracey, 2020). *Second*, compared to pure OL, BL stimulates students’ interest and attention more effectively (L. Ma & Lee, 2021). *Third*, in online class, students often lack a sense of community because they are detached from the interpersonal interaction in F2F learning (Dennis, 2020). *Fourth*, BL facilitates students to study at their speed and enables students to put theory into practice. *Fifth*, the community of inquiry is associated with student learning and satisfaction (Martin et al., 2022b), and the teaching and social presence of BL is higher than OL (Harrell & Wendt, 2019). *Sixth*, according to the SDT, BL may meet learners’ competence and relatedness needs better than OL. In addition, BL can better foster student deep learning than OL (X. Y. Wu, 2024).

There is a significant difference in BL’s effects on learning outcomes. Specifically, BL has upper-medium effects on affective outcomes and cognitive outcomes and an insignificant effect on behavioral outcomes. The result is partially consistent with those of Li and Wang (2022). A recent second-order meta-analysis reveals that OL has much better effects on behavior outcomes than affective and cognitive outcomes (Martin et al., 2022a). Based on this, the behavioral outcomes are the lowest when comparing the effects of blended and online learning.

### 5.2. Moderator Analysis (RQ2)

#### 5.2.1. Class Size

It has a moderating impact on BL. Specifically, BL has upper-medium effects when the class size is 31–50 and ≤30, a lower-medium effect when the class size is 51–100, and an insignificant effect when the class size is >100. This result is consistent with the findings of Li and Wang (2022) and Tutal and Yazar (2021). Moreover, the effectiveness of BL will be reduced when the class size is too large (i.e., >100). This is because teachers are essential in BL, but excessive class sizes will reduce the efficiency of teachers’ teaching management (Broadbent, 2017). As a result, teachers may face great challenges in managing and conducting BL. In other words, smaller class sizes are more beneficial for student learning (Glass & Smith, 1979; Li & Wang, 2022; Shin & Chung, 2009).

#### 5.2.2. Grade Level

The grade level moderates BL’s effects, which is consistent with the findings of Güler et al. (2023) and Tutal and Yazar (2021). Specifically, BL’s effects on K-12 are large, are upper-medium on university, and are small on adults. Usually, OL has a much better effect on university students than K-12 students (Martin et al., 2022a). OL requires higher levels of self-regulated and self-directed abilities than BL (Xu et al., 2023), which students in grades K-12 lack. Meanwhile, BL is beneficial for improving students’ self-regulated and self-directed abilities (Ruchan & Adem, 2018). So, BL’s effect on the K-12 level is the best. Moreover, adult students often need to balance work, life, and learning, making it difficult to involve them in BL (Romero, 2011). However, due to the flexibility of BL, it still positively affects adult students’ learning.

#### 5.2.3. Learning Duration

It is an insignificant moderator. Specifically, <1 month has a large effect, and 1–3 months and ≥3 months have upper-medium effects. The result is in line with those of Li and Wang (2022), Tutal and Yazar (2021), Vo et al. (2017), and Yu et al. (2025). Longer durations will narrow the effects of BL compared to OL; that is, longer duration will result in smaller BL effects. It may be interpreted that longer durations with increased on-screen time will increase stress and anxiety and generate burnout and exhaustion (Mheidly et al., 2020). Past meta-analyses also find that the effects of BL and OL decline with durations that are too long (i.e., 1 semester) (Martin et al., 2021; Vo et al., 2017). Moreover, in most BL, the proportion of F2F is higher than OL (*N* = 31, POL ≤ 50%) and the impact of OL is likely to increase with familiarity and long-term exposure.

#### 5.2.4. Subject

It is a significant moderator. Specifically, the effect of non-STEM is large and STEM is lower-medium. The result is consistent with those of Låg and Sæle (2019) and Yu et al. (2025), but not with Vo et al. (2017). Typically, STEM subjects tend to be more challenging and difficult than non-STEM subjects (Thomas, 1990). In BL, non-STEM students perceive their learning more positively than STEM students (Owston et al., 2020). Accordingly, BL has a larger impact on enhancing non-STEM subjects than STEM subjects.

#### 5.2.5. Teacher

The teacher has a moderating impact. The ES of different teachers is larger than that of the same teachers, which is consistent with the results of Vo et al. (2017) but inconsistent with Li and Wang (2022) and Means et al. (2013). Teacher quality matters most in influencing student learning (Goldhaber, 2016). As differences exist among teachers, such as different teaching expertise, styles, abilities, and digital literacy, this result in different teaching outcomes (Darling-Hammond, 2000; Li & Wang, 2022). In educational settings, teachers are often different. Differences may bring potential merits. In addition, the same teachers in BL and OL can better reflect their actual effects on students’ learning. This research reveals that BL still has a lower-middle ES when taught by the same teachers. That is, whether teachers are the same or not, BL is more effective for enhancing student learning than OL.

#### 5.2.6. POL

The POL significantly moderates the effects of BL, which is consistent with Yu et al. (2025). Specifically, 50% of POL has the largest effect, followed by 30–49% and 51–69% (upper-medium effect), and 70–79% of POL has small effects, while 80% of POL has an insignificant effect. The result is in line with those of Owston and York (2018) and Tambunan et al. (2021). POL is related to BL’s flexibility and successful implementation (Boelens et al., 2017; Yudt et al., 2024). Low or high POL may decrease students’ sense of community and basic psychological needs and reduce their learning effects. In particular, POL is the most significant indicator distinguishing BL from OL.

#### 5.2.7. TOI

TOI is an insignificant moderator. Specifically, the effect of mixed interaction is large, synchronous interaction is upper-medium, and asynchronous interaction is upper-small. The result is consistent with those of Li and Wang (2022) and Yu et al. (2025). Asynchronous interaction can increase flexibility by providing flexible learning time (Moradimokhles & Hwang, 2022). However, inappropriate asynchronous interactions were less effective (Zhu et al., 2021), e.g., delayed feedback and communication may reduce student enthusiasm, interest, and relatedness. Synchronous interactions motivate students and improve their sense of belonging and performance (Zhu et al., 2021). Students value real-time interactions with peers and teachers (Zhu et al., 2021). Synchronous interaction surpasses asynchronous interaction in lower-difficulty subjects and vice versa (Van der Kleij et al., 2015). Thus, a mix of asynchronous and synchronous is regarded as the optimal way to satisfy students’ basic psychological needs and support their learning online (Moorhouse & Wong, 2022).

#### 5.2.8. OGA

OGA is a significant moderator. Specifically, mixed learning has a large effect, group learning has an upper-medium effect, and independent learning has a lower-middle effect, partially consistent with the findings of Li and Wang (2022) and Yu et al. (2025). Unlike prior meta-analyses (Bernard et al., 2014; Li & Wang, 2022; Means et al., 2013; Vo et al., 2017), this meta-analysis reveals that independent learning is still beneficial for BL (Yu et al., 2025). Online class students often complain of isolation (Dennis, 2020). They may lack a sense of belonging and have more workload than learners in BL (D. H. Lim et al., 2007). Group learning is a particular type of collaborative learning (N. T. T. Thai et al., 2020), and it is better than individual learning (Chen et al., 2018). For instance, groups could achieve a higher sense of belonging and solve problems better than individuals could (Alonso et al., 2010). Group learning also meets learners’ relatedness needs.

#### 5.2.9. Region

The region is a significant moderator, which is consistent with the results of Li and Wang (2022), Schmid et al. (2023), and Yu et al. (2025). Specifically, BL has a large impact on Asia, a lower-medium impact on Australia, and upper-small impacts on European and North American students. Learner characteristics are an important factor that cannot be neglected when designing BL. Different regions often have diverse cultures (e.g., collectivist and individualist). Cultural differences may result in different adoptions of OL (Zhao et al., 2021) and different learning outcomes (Güler et al., 2023). Moreover, in the digital era, regional economic status and digital level may vary. These factors can influence BL’s effects and student performance. Particularly, disparities in the number of ES across different regions reveal the potential digital educational inequality worldwide. In addition, due to the limited number of ES in Australia (*N* = 1), its result should be viewed cautiously.

#### 5.2.10. Publication Type and Year

The publication type is an insignificant moderator. Specifically, the effects of dissertations are large, journals are upper-medium, and conferences are insignificant. Different publication types may have different preferences, e.g., journal articles tend to report larger effect sizes (Cheng et al., 2019; Martin et al., 2021; Vo et al., 2017). Overall, publication type is not one cause of heterogeneity.

The publication year is an insignificant moderator, which is consistent with the findings of Yu et al. (2025). BL’s effects slightly rise with the years’ increase. The result could be interpreted with the opinion of Edgerton (2010) and technological diffusion (Rogers, 2003). BL and OL will continue to evolve and improve, and students and teachers are becoming increasingly familiar with BL. Thus, students’ learning experience and BL’s effects will also be better. In summary, BL’s effects are larger than those of OL over the years.

### 5.3. Suggestions for Educators and Researchers

This study compares BL’s effects with OL and examines 11 moderators’ influences. Though there are some limitations, e.g., not all terms on BL or OL are used, some valuable findings enlighten suggestions for future BL practices and research.

(1) This meta-analysis suggests that BL is more effective in improving students’ learning performance than OL, especially in promoting students’ affective and cognitive outcomes. Moreover, the effects on behavioral outcomes are the same between BL and OL. So, compared to OL, BL is suggested for promoting students’ cognitive (e.g., academic achievement, critical thinking, etc.) and affective outcomes (e.g., learning motivation, attitude, etc.). Given the limited number of studies included and the significant heterogeneity present, the results should be treated cautiously, and more research should be conducted in the future to improve the quality and robustness of future meta-analysis.
(2) Based on the finding of this study, maintaining a reasonable class size is important to improve BL’s effectiveness. Here, 50 or less is the suggested class size. Meanwhile, future researchers can pay more attention to BL’s effects in large classes, i.e., >100 (*N* = 4).
(3) Based on the findings of this study, BL can promote all stages of students’ learning compared to OL. However, BL is more effective for K-12 and universities. Given that the number of ES in adult students (*N* = 2) and K-12 (*N* = 5) is far less than in university (*N* = 59), more studies should be performed in the future.
(4) This meta-analysis suggests that though different durations are all effective, durations 3 months or less are suggested. Moreover, future researchers can pay more attention to the effects of durations of <1 month (*N* = 4).
(5) Based on the findings of this study, BL can be used to promote student learning in both STEM and non-STEM subjects, with greater potential for non-STEM subjects. In addition, future researchers can make more detailed divisions in subjects (e.g., discipline areas) to reveal more findings.
(6) Based on the findings of this study, even with the same teacher, BL still outperforms OL in promoting student learning. Namely, teacher consistency confirms that BL is more effective than pure OL. Moreover, teacher inconsistency may bring some merits but can also deepen inequity in education, and there is a need to continuously optimize teacher professional development and improve teacher competence in the future.
(7) This meta-analysis suggests that POL is the critical factor that influences BL’s effects (significant moderator). Teachers should select appropriate POL to optimize BL design and students’ experiences. This study reveals that 50% POL is best, followed by 30–49% and 51–69%, which are suggested. Moreover, future researchers could do more experiments to test the effects of 70–79% (*N* = 2) and 80% (*N* = 1).
(8) Based on the findings of this study, both asynchronous and synchronous online interactions are effective in BL, while mixed interaction (asynchronous + synchronous) is more effective. Teachers should use their advantages to maximize BL’s effects according to specific subjects and contents. Synchronous interaction may be more effective in low-difficulty subjects and content, but asynchronous interaction is more effective in some difficult subjects and content (Van der Kleij et al., 2015). Combining asynchronous and synchronous interactions to achieve better teaching effects is best.
(9) Based on the findings of this study, educators should take advantage of group and independent learning to optimize student learning in BL. Mixed learning combines group and individual learning to maximize BL’s effectiveness. Given that the number of ES in group learning (*N* = 3) is limited, future research could explore more.
(10) Based on the findings of this study, compared to OL, BL can enhance Asian, European, North American, and Australian students’ learning. In addition, regional analysis also reveals the potential digital divide and inequity in the world. Future research should explore BL’s effectiveness in more diverse regions or countries, e.g., Australia, South America, Africa, etc.

## 6. Conclusions

### 6.1. Major Findings

This meta-analysis compares the effects of BL and OL. The result suggests that BL’s effect is upper-medium compared to OL; namely, BL is more effective than OL. Meanwhile, the class size, grade level, subject, teacher, POL, TOI, OGA, and region moderate the effect of BL. Namely, these moderators are causes of homogeneity. Moreover, this meta-analysis shows that BL will achieve better effects than pure OL (1) on affective and cognitive outcomes (2) for a class size of 0~50 students (3) on K-12 and university students (4) within 3 months of intervention (5) on non-STEM subjects (6) with different teachers (7) with 30~69% POL (8) using mixed interaction (9) with mixed and group learning (10) on Asian students. Last, due to some limitations in this study, the findings should be viewed critically.

### 6.2. Major Limitations

Despite the valuable findings of the present research, some limitations are as follows. First, the included articles are only published in English, and other languages are excluded. Moreover, due to the existence of some uncommon terms for BL and OL, certain terms may be missing in literature searches (e.g., mixed mode learning, internet-based instruction, etc.), and future research could incorporate them into searches. Second, some subgroups are not large, e.g., 80% (*N* = 1), Australia (*N* = 1), and so on. Third, the heterogeneity is considerable; there may be some potential moderating variables that were missed, e.g., test and knowledge types, etc. These limitations provide directions for future research. Fourth, as technology is developing and BL and OL will evolve, more experimental studies should be conducted to explore their effects. In addition, some technologies combined with BL and OL should also be examined, e.g., future researchers can explore the impact of AI-assisted BL and OL (R. Wu & Yu, 2024); online simulation is also an important direction (Selcuk et al., 2025).

## Figures and Tables

**Table 1:** Inclusion and exclusion criteria.

No.	Included Criteria	Excluded Criteria
(1)	It should compare the effect of BL with OL.	No comparison of BL and OL.
(2)	Published in English.	Languages other than English.
(3)	Experimental or quasi-experimental study.	Non-experimental.
(4)	It includes the required information, e.g., sample size, mean, standard deviation, *t*- and *p*-value, and other related data.	Lack of necessary data to calculate ES, such as sample size.

**Table 2:** Coding scheme.

Categories	Variables	Subtypes	Description
Dependent variable	Learning outcome	Cognitive, affective, and behavior outcomes	Cognitive outcomes = academic achievement, thinking skills, etc. Affective = motivation, satisfaction, etc. Behavioral outcomes = practical skills, etc.
Background features	Class size	≤30, 31–50, 51–100, and >100	Number of participants in the control and experimental group.
	Grade level	K-12, university, and adult	Grade level of participants.
	Learning duration	<1, 1–3, and ≥3 (months)	Duration of intervention.
	Subject	STEM, non-STEM	STEM = science, technology, engineering, and math. Non-STEM = disciplines other than STEM, e.g., humanities and arts.
	Teacher	Same and different	Comparison between teachers in the experimental and control group.
	Region	Asia, Europe, and North America	Areas where interventions take place.
BL design variables	POL	30–49%, 50%, 51–69%, 70–79%, and 80%	Percentage of total learning time spent on OL.
	TOI	Synchronous, asynchronous, and mixed interaction (synchronous + asynchronous)	The type of online communication.
	OGA	Group, independent, and mixed learning (group + independent)	The type of OL activity. Independent learning = self-learning.
Literature features	Publication year	Integers: 2006 to 2024	The published year of the literature.
	Publication type	Journals, dissertations, and conferences	The published type of the literature.

**Table 3:** Overall ES of BL and heterogeneity test.

*N*	*SMD*	95% *CI*		2-Tail Test		Heterogeneity		
		Lower Limit (LL)	Upper Limit (UL)	*Z*	*p*	*Q*	*I* ^2^	*p*
67	0.611	0.466	0.755	8.288	0.000	549.844	87.997	0.000

**Table 4:** BL’s effects on different types of learning outcomes.

Learning Outcome	*N*	*SMD*	95% *CI*		*Z*	*p*	*Q*-Between
Cognitive outcomes	45	0.698	0.509	0.887	7.253	0.000	*Q* = 16.333 ***
Affective outcomes	17	0.533	0.271	0.795	3.984	0.000	
Behavioral outcomes	5	0.140	−0.059	0.339	1.375	0.169	

*Note:* *** *p* < 0.001.

**Table 5:** BL’s effects on varying class sizes, grade levels, durations, subjects, and teachers.

Moderators	*N*	*SMD*	95% *CI*		*Z*	*p*	*Q*-Between
**Class size**							
≤30	28	0.659	0.461	0.858	6.509	0.000	*Q* = 27.402 ***
31–50	23	0.744	0.423	1.066	4.543	0.000	
51–100	12	0.447	0.152	0.742	2.970	0.003	
>100	4	0.097	−0.046	0.241	1.331	0.183	
**Grade level**							
K-12	5	0.885	0.355	1.416	3.270	0.001	*Q* = 19.681 ***
University	59	0.609	0.445	0.773	7.296	0.000	
Adult	2	0.212	0.097	0.326	3.624	0.000	
**Duration**							
<1	4	0.799	0.492	1.106	5.101	0.000	*Q* = 6.388
1–3	28	0.569	0.404	0.735	6.739	0.000	
≥3	32	0.529	0.273	0.786	4.039	0.000	
**Subject**							
Non-STEM	21	1.025	0.720	1.329	6.602	0.000	*Q* = 12.570 ***
STEM	46	0.421	0.284	0.558	6.014	0.000	
**Teacher**							
Different	11	1.181	0.702	1.661	4.826	0.000	*Q* = 9.851 **
Same	37	0.410	0.236	0.583	4.621	0.000	

*Note*: *** *p* < 0.001, ** *p* < 0.01.

**Table 6:** BL’s effects on diverse POL, TOI, OGA, regions, and publication types.

Moderators	*N*	*SMD*	95% *CI*		*Z*	*p*	*Q*-Between
**POL**							
30–49%	15	0.732	0.302	1.162	3.336	0.001	*Q* = 25.116 ***
=50%	16	0.749	0.460	1.038	5.076	0.000	
51–69%	6	0.610	0.424	0.797	6.407	0.000	
70–79%	2	0.212	0.097	0.326	3.624	0.000	
=80%	1	0.162	−0.338	0.661	0.634	0.526	
**TOI**							
Mixed	10	1.343	0.851	1.835	5.352	0.000	*Q* = 15.107 **
Synchronous	7	0.664	0.121	1.208	2.396	0.017	
Asynchronous	37	0.384	0.237	0.531	5.129	0.000	
**OGA**							
Group	3	0.720	0.213	1.228	2.782	0.005	*Q* = 7.905 *
Independent	28	0.403	0.260	0.546	5.532	0.000	
Mixed	7	0.868	0.473	1.264	4.302	0.000	
**Region**							
Asia	38	0.815	0.601	1.028	7.482	0.000	*Q* = 11.240 *
North America	21	0.328	0.104	0.553	2.864	0.004	
Europe	7	0.373	0.069	0.676	2.408	0.016	
Australia	1	0.425	0.039	0.811	2.158	0.031	
**Publication**							
Journals	50	0.636	0.475	0.797	7.749	0.000	*Q* = 5.507
Dissertations	11	0.780	0.430	1.131	4.359	0.000	
Conferences	6	0.121	-0.437	0.679	0.425	0.671	

*Note*: *** *p* < 0.001, ** *p* < 0.01, * *p* < 0.05.

## Data Availability

The original contributions presented in this study are included in the article. Further inquiries can be directed to the corresponding authors.

## Abbreviations

The following abbreviations are used in this manuscript:

BL	Blended learning
OL	Online learning
F2F	Face-to-face
SDT	Self-determination theory
POL	Proportion of online learning
TOI	Type of online interaction
OGA	Online learning activity
